# Exploring the Adoption of Mobile Health Apps Among Patients with Head and Neck Cancer After the COVID-19 Pandemic: Secondary Analysis of Cross-Sectional Survey

**DOI:** 10.2196/65192

**Published:** 2026-04-15

**Authors:** Christian Wilhelm, Matthias Scheich, Michael Winter, Johannes Allgaier, Johanna Rippstein, Sylke Ruth Zeissig, Rüdiger Pryss, Carsten Vogel, Johannes Schobel, Katrin Radeloff, Joanna Karolonek, Agmal Scherzad, Stephan Hackenberg, Rudolf Hagen

**Affiliations:** 1 Department of Otorhinolaryngology, Plastic, Aesthetic and Reconstructive Head and Neck Surgery, Julius Maximilian University of Wuerzburg, Schneider-Strasse 11, Wuerzburg, 97080, Germany, 49 931-201-21323, 49 931-201-21321; 2 University of Würzburg, Würzburg, Germany; 3 Institute for Medical Data Sciences, University of Wuerzburg, Wuerzburg, Germany; 4 Institute for Clinical Epidemiology and Biometry, University of Wuerzburg, Wuerzburg, Germany; 5 Regional Centre Wuerzburg, Bavarian Cancer Registry, Bavarian Health and Food Safety Authority, Wuerzburg, Germany; 6 DigiHealth Institute, Neu-Ulm University of Applied Sciences, Neu-Ulm, Germany; 7 Department of Otorhinolaryngology, Plastic, Aesthetic and Reconstructive Head and Neck Surgery, Evangelisches Krankenhaus Oldenburg, Medical Campus of the Carl von Ossietzky University of Oldenburg, Oldenburg, Germany

**Keywords:** head and neck cancer, COVID-19 pandemic, mHealth, digital health solutions, cancer care, telemedicine, digital literacy, health care access, patient-centered care, mobile health

## Abstract

**Background:**

Patients with head and neck cancer (HNC) face significant challenges in accessing coordinated care due to the complexity and multimodality of their treatment and the impact on vital functions. The COVID-19 pandemic has disrupted cancer care while accelerating the adoption of digital health solutions. Mobile health (mHealth) apps offer potential solutions for remote symptom monitoring, communication between patients and providers, and continuity of care. Nevertheless, their acceptance among patients with HNC remains limited due to age-related digital divides and concerns about accessibility.

**Objective:**

The aim of this study was to investigate the impact of the COVID-19 pandemic on patients with HNC and to explore their attitudes toward mHealth apps as a supplement to cancer treatment.

**Methods:**

A secondary analysis of a cross-sectional survey was conducted between January 2023 and May 2024 at Julius Maximilian University of Würzburg in Germany. A total of 355 patients with HNC were recruited and completed the structured “Cancer and COVID-19” survey via the Corona Health app or in paper form. The 25-question survey assessed sociodemographic information, the impact of the pandemic on diagnosis/treatment/aftercare, and interest in mHealth apps. Descriptive statistics and bivariate correlations were used for the analysis.

**Results:**

The cohort comprised 261 (74%) men and 94 (26%) women with an average age of 67 (SD 11.5) years. Most participants (n=264, 74%) stated that the pandemic had no impact on their cancer treatment, although 20% (n=71) experienced disruptions, particularly in follow-up appointments and treatment monitoring. Only 10% (n=36) currently used health apps, but 57% (n=203) expressed a willingness to use mHealth technologies. Younger patients, patients with higher education, and participants who were more affected by the pandemic showed greater openness to digital health solutions. The most significant barriers included age, digital literacy, and perceived usefulness, while preferred app features included interaction with physicians (n=160, 45%) and data sharing with researchers (n=153, 43%).

**Conclusions:**

Although the COVID-19 pandemic had only a limited direct impact on HNC care at the institution at hand, it revealed significant patient interest in mHealth apps. However, significant barriers remain, particularly among older adults with lower digital literacy. Future mHealth initiatives should focus on improving digital literacy, addressing privacy concerns, demonstrating clinical benefits, and developing personalized, accessible solutions to optimize cancer care for this vulnerable population.

## Introduction

Head and neck cancer (HNC) is a particularly difficult form of cancer due to its impact on vital functions, such as speaking, swallowing, and breathing, as well as its demanding, multimodal treatment pathways [1]. The treatment of these patients requires continuous medical, psychosocial, and logistical support throughout all phases of care. Ensuring continuity of care and patient retention is therefore a key objective in head and neck oncology [2].

Despite advances in treatment, many patients with HNC face barriers to accessing timely and coordinated care [3]. Advanced age, multiple comorbidities, and the physical and emotional burden of the disease can limit patients’ autonomy and complicate long-term follow-up care [4]. In addition, patients often report unmet needs in terms of communication, symptom management, and psychological support—highlighting gaps in traditional care structures [5].

Digital health technologies, particularly mobile health (mHealth) apps, offer new tools to bridge these gaps [6]. By supporting remote symptom monitoring, improving communication between patients and providers, and enabling self-management, mHealth can improve both the quality of care and patient autonomy [7-10]. However, acceptance among patients with HNC remains limited, partly due to age-related digital divides and a lack of tailored, accessible solutions [11,12].

The COVID-19 pandemic posed significant challenges to oncology care, including appointment postponements and treatment interruptions [13,14]. Simultaneously, it accelerated the adoption of telemedicine and digital health tools in many health care systems. For patients with HNC, the pandemic provided a unique opportunity to try out digital solutions—not just as an emergency measure, but as a permanent component of cancer care [15].

This study examines the experiences of patients with HNC during the COVID-19 pandemic and their perception of mHealth apps. Using self-reported patient data from the Corona Health app, we analyze disruptions in cancer treatment, patterns of digital health service use, and the most important factors that promote or hinder the adoption of mHealth. The results may, in turn, promote the development of accessible, patient-centered digital interventions for people with HNC.

## Methods

### Study Design and Participants

This study is a secondary analysis of data collected via the Corona Health mobile app platform, a study- and sensor-based mHealth infrastructure developed to investigate various aspects of the COVID-19 pandemic and conduct multiple questionnaire-based studies [16]. Details on the technical implementation of the platform, data collection procedures, and characteristics of the initial cohort have been published by Beierle et al [16,17] (2021). For this analysis, we used cross-sectional data from patients with HNC, who participated through a dedicated study module integrated into the Corona Health app. Participants were recruited between January 2023 and May 2024 at the Department of Otorhinolaryngology, Plastic, Aesthetic, and Reconstructive Head and Neck Surgery at Julius Maximilian University of Würzburg, Germany. Recruitment took place primarily within the department’s clinical infrastructure. The weekly tumor follow-up clinic for patients with HNC, which offers both initial consultations and follow-up appointments, was the main recruitment site, with approximately 35 to 40 patients participating per week. During waiting times, suitable patients were informed about the study and invited to participate. In addition, inpatients from 2 wards specializing in the diagnostic and therapeutic treatment of HNC were approached to participate. Patients with a confirmed HNC diagnosis were included in the study, whereas patients with cognitive impairments or language barriers that prevented participation were excluded.

**Figure 1.**
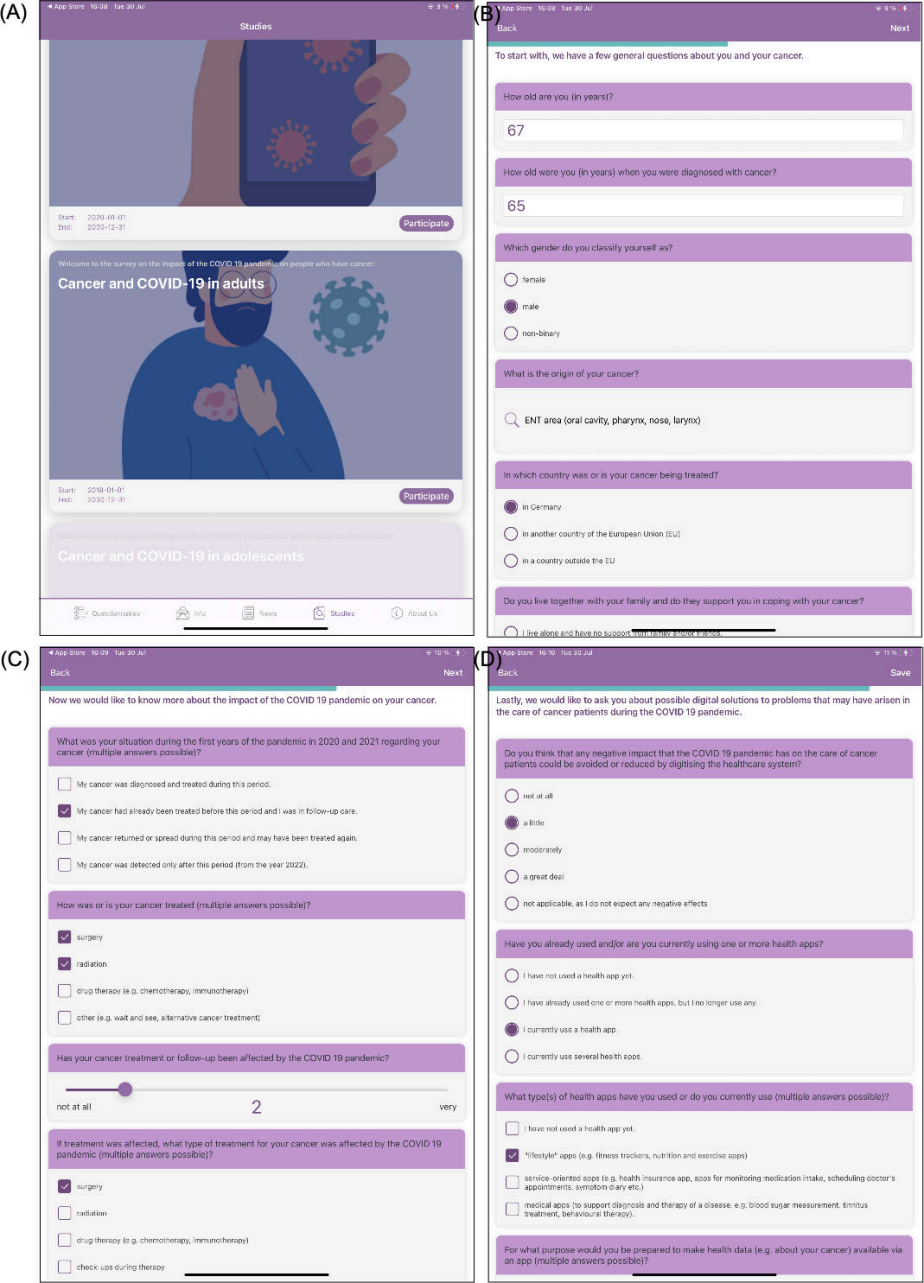
iOS in-app screenshots. (A) Overview of studies with the cancer and COVID-19 survey. (B) Part A of the survey: sociodemographic information. (C) Part B of the survey: the impact of the pandemic on cancer. (D) Part C of the survey: mobile health app use.

Participants who were unable or unwilling to use the app completed a paper version of the same survey (see Figure 1), the responses to which were later digitized and entered into the database. The survey comprised 25 specially designed questions (Q 1‐25) on participants’ sociodemographic characteristics (Part A); the impact of the pandemic on diagnosis, treatment, and follow-up care (Part B); and their interest and willingness to use mHealth apps (Part C).

The response formats included single choice (Q 1‐9, Q 20‐22, Q 24), multiple choice (Q 10‐11, Q 13, F 22‐23, F 25), and matrix/rating scale questions (F 12, F 14‐19) with 1 to 8 points to reduce central tendency bias. For analysis, scores were divided into 4 categories (1, 2-4, 5-7, 8) and labeled according to context (eg, never, rarely, sometimes, and often for F 16‐17; no, low, moderate, and high for F 12, F 14‐15). Trained health care professionals assisted participants in completing the survey.

The survey was developed by experts in epidemiology and ear, nose, and throat medicine as well as head and neck surgery at the University Hospital of Würzburg, Germany. Before large-scale use, it was tested in 3 oncology outpatient clinics with approximately 30 patients, who evaluated the clarity and relevance of the individual questions. Based on their feedback, questions were refined, added, or removed to improve comprehensibility and validity. This iterative process resulted in a robust, patient-centered instrument that enabled a meaningful assessment of the experiences and perspectives of patients with HNC during the pandemic. The study was also open to anyone who could download the app for free from the Google and Apple app stores.

### Data Analysis

Survey responses were analyzed to identify patterns and significant findings related to the impact of the COVID-19 pandemic on HNC treatment and attitudes toward mHealth apps. Descriptive statistics were used to summarize demographic characteristics and survey items.

The relationships between demographic variables and survey variables were examined using Pearson correlation for continuous data and Spearman ρ for ordinal data. Statistical significance was set at *P*≤.05, with interpretation emphasizing the strength and direction of correlations. Coefficients of approximately |0.10‐0.29|, |0.30‐0.49|, and ≥|0.50| were interpreted as weak, moderate, and strong associations, respectively [18]. Positive coefficients indicated that the variables increased together, whereas negative coefficients indicated inverse relationships.

The study was exploratory in nature and aimed to characterize the experiences of patients with HNC during the pandemic and their attitudes toward the use of mHealth. Due to the lack of previous data on mHealth acceptance in this population, formal performance calculations were not possible. The final sample of 355 participants (response rate: 60%) exceeded the typical minimum requirements for correlation analyses (30‐50 participants per variable) and provided sufficient power (>80%) to detect medium effect sizes (*r*≥0.15) at α=.05. Subgroup analyses among mHealth users (34/355, 10%) were found to be undersized, and the corresponding results should be interpreted as preliminary and validated in larger samples.

### Ethical Considerations

The Corona Health app used was developed and implemented in accordance with medical device regulations and received the appropriate certification [19]. The University of Würzburg ethics committee approved the original data collection for this study (130/20-me), with explicit coverage for both initial collection and future secondary analyses of the data. The primary study describing the Corona Health platform and its data collection across multiple integrated studies has been published by Beierle et al [16]. Therefore, no additional institutional review board approval was required for this secondary analysis. Under local regulations and institutional guidelines [20], the secondary use of existing anonymized data does not require separate ethical review. All procedures complied with institutional ethical standards for research involving human subjects, and participant data were completely anonymized to ensure privacy protection. Participation was voluntary and no financial compensation was provided. Finally, this cross-sectional study followed the STROBE (Strengthening the Reporting of Observational Studies in Epidemiology) guidelines for reporting cross-sectional studies (Checklist 1).

## Results

The results are divided into 3 main sections according to the survey structure: (1) demographic data and clinical characteristics of participants, (2) impact of the pandemic on cancer care, and (3) attitudes toward mHealth apps.

### Part A: Participant Sociodemographics

A total of 355 individuals with head and neck malignant neoplasms participated in the study, including 261 (74%) males and 94 (26%) females, with a median age of 67 years (IQR 18‐92 y; Q 1 and Q 3) at the time of the survey.

The median age at cancer diagnosis was 65 (IQR 55-70) years (Q 3). Time since diagnosis was calculated by subtracting age at diagnosis (Q 3) from the current age (Q 2). In addition, 80% (n=283) of the participants received their initial diagnosis within the past 5 years, with the proportion decreasing from 36% (n=102) diagnosed within the past year to 8% diagnosed 5 years ago.

All individuals received their treatment in Germany (Q 5). Additionally, 18% (n=65) of the participants had a history of other cancers, mainly gastrointestinal cancer (n=18, 5%), malignant melanoma (n=10, 3%), and blood or lymphatic cancer (n=10, 3%).

Half of the participants (n=179, 50%) had 8 to 9 years of school education, 158 (45%) had a higher educational level, and 18 (5%) had a lower educational level.

More than half of the participants (n=192, 54%) had completed 3 to 4 years of professional training or higher education. About a quarter (n=92, 26%) had a longer qualification, while a fifth (n=71, 20%) had a shorter qualification.

Approximately 3 quarters (n=261, 74%) of the participants live with and receive support from their families. One in 5 (n=72, 20%) participants lives alone but receives support from family and/or friends. A small number of individuals either live alone without support (n=16, 5%) or live with their families but receive little or no support (n=6, 2%).

At the time of the survey, approximately 1 out of 4 (n=90, 25%) participants was employed, while two-thirds (n=239, 68%) were unemployed. Of those who were unemployed, the majority were either doing housework and/or raising children, retired, or preretired (n=233, 66%), with a small minority actively seeking work (n=6, 2%).

In summary, a comprehensive summary of all results for Part A can be found in Table 1.

**Table 1.** Participant demographics and clinical characteristics (see Table S1 in Multimedia Appendix 1 for the detailed overview of the questions).

Variable and category	Values, n (%)
Age by gender	
20-29	Female: 4 (1.1) Male: 0 (0)
30‐39	Female: 7 (2) Male: 2 (0.6)
40‐49	Female: 4 (1.1) Male: 8 (2.3)
50-59	Female: 15 (4.2) Male: 40 (11.3)
60‐69	Female: 34 (9.6) Male: 106 (29.9)
70‐79	Female: 21 (5.9) Male: 77 (21.7)
80‐89	Female: 8 (2.3) Male: 27 (7.6)
90‐99	Female: 1 (0.3) Male: 1 (0.3)
Total	Female: 94 (26) Male: 261 (74)
Time between survey and diagnosis (y)	
0	102 (28.7)
1	59 (16.6)
2	43 (12.1)
3	34 (9.6)
4	23 (6.5)
5	22 (6.2)
>5	72 (20.3)
Cancer entities (ie, origin of cancer)	
Renal	1 (0.3)
Urologic	3 (0.8)
Gynecologic	4 (1.1)
Lung	6 (1.7)
Blood/lymphatic	10 (2.8)
Melanoma	10 (2.8)
Other cancer	13 (3.7)
Gastrointestinal tract	18 (5.1)
HNC^a^ only	290 (81.7)
School education (y)	
≤7	18 (5.1)
8-9	179 (50.4)
10	73 (20.6)
11-12	42 (11.8)
≥13	43 (12.1)
Job qualification (y)	
0	32 (9)
1-2	39 (11)
3-4	192 (54.1)
5-6	47 (13.2)
≥7	45 (12.7)
Living situation	
Alone, no support	16 (4.5)
Alone, supported by family/friends	72 (20.3)
With family, supported	261 (73.5)
With family, little/no support	6 (1.7)
Employment status	
Retired	233 (65.6)
Seeking work	6 (1.7)
Civil servant	79 (22.3)
Self-employed	11 (3.1)
Other	26 (7.3)

a HNC: head and neck cancer.

### Part B: Impact of the COVID-19 Pandemic

Among 355 participants, 118 (33%) were diagnosed with cancer before the pandemic (2019 and earlier), 108 (30%) during the pandemic (2020 and 2021), and 126 (35%) of them after the pandemic (2022 and later). Of all respondents, only 13 stated that they had been treated due to a recurrence of their cancer.

Out of 355 participants, 290 (82%) underwent surgery as part of their treatment plan. Radiation therapy was included for 222 (63%) participants, and drug therapy (eg, chemotherapy and immunotherapy) was part of the treatment for 126 (35%) participants. Additionally, 11 (3%) participants received other therapies (eg, watchful waiting and alternative cancer treatments). Combination treatments were common: 170 (48%) participants had both surgery and radiation, 112 (32%) had both radiation and drug therapy, 111 (31%) had surgery only, 14 (4%) had radiation only, 5 (1%) had drug therapy only, and 7 (2%) received solely other therapies.

About 3 out of 4 participants reported no impact of the pandemic on their therapy and aftercare (n=264, 74%), while most of those who experienced disruptions considered them to be minor (n=57, 16%).

Out of 355 participants, 285 (80%) did not report any disruption in their therapy or aftercare. Among the remaining 70 (20%) participants, the most documented disruptions were in follow-up examinations (34, 10%) and checkups during therapy (21, 6%). Fewer participants experienced disruptions in radiation therapy (15, 4%), surgery (14, 4%), drug therapy (10, 3%), and supportive therapy (9, 3%).

More than half of the participants reported no fear of SARS-CoV-2 infection during (n=204, 57%) or after the pandemic (n=205, 58%). Approximately 1 in 4 participants experienced little fear during (n=94, 26%) and slightly more after the pandemic (n=115, 32%). Moderate fear declined from 11% (n=39) during the pandemic to 7% (n=24) after the pandemic, as did very strong fear (n=18, 5% during; n=11, 3% after).

Most participants did not experience cancellations of appointments by the clinic due to the pandemic (n=299, 84%), and even fewer patients canceled appointments themselves (n=309, 87%). About 1 in 10 participants had appointments rarely canceled by the clinic (n=47, 13%) and rarely canceled appointments themselves (n=40, 11%). Almost no participants faced regular appointment cancellations.

Around two-thirds (n=223, 63%) of the participants had no psychological effects due to the pandemic at all, 101 (28%) participants had minor effects, and only 26 (7%) and 5 (1%) of them were affected moderately or highly.

Similarly, around two-thirds of the participants had no fear of negative effects of the pandemic on their own cancer therapy (n=234, 66%), 102 (29%) participants had minor fear, while 16 (5%) and 3 (1%) documented moderate or major fear, respectively.

In summary, a comprehensive summary of all results for Part B can be found in Table 2.

**Table 2.** Treatment characteristics and COVID-19 pandemic impact (see Table S1 in Multimedia Appendix 1 for detailed overview of the questions).

Variable and category	Values, n (%)
Date of cancer diagnosis	
2019 and earlier	118 (33.5)
2020‐2021	108 (30.7)
2022 and later	126 (35.8)
Type of therapy	
Surgery	290 (81.7)
Radiation	222 (62.5)
Drug therapy	126 (35.5)
Other	11 (3.1)
Surgery only	111 (31.3)
Radiation only	14 (3.9)
Drug therapy only	5 (1.4)
Other only	7 (2)
Surgery+radiation	170 (47.9)
Radiation+drug therapy	112 (31.5)
Impact on therapy and aftercare	
None	264 (74)
Minor	57 (16)
Moderate	28 (8)
Major	6 (2)
Affected type of therapy (n=70/355)	
Supportive therapy	9 (2.5)
Drug therapy	10 (2.8)
Surgery	14 (3.9)
Radiation	15 (4.2)
Therapy checkups	21 (5.9)
Follow-up	34 (9.6)
Not affected	285 (80.3)
Fear of SARS-CoV-2 infection	
During the pandemic	
No	204 (57.7)
Minor	94 (26.5)
Moderate	39 (11)
Major	18 (5.1)
After the pandemic	
No	205 (57.7)
Minor	115 (32.4)
Moderate	24 (6.8)
Major	11 (3.1)
Cancellation of appointments	
Clinic	
Never	299 (84.2)
Rarely	47 (13.2)
Sometimes	5 (1.4)
Frequently	4 (1.1)
Patient	
Never	309 (87)
Rarely	49 (11.3)
Sometimes	6 (1.7)
Frequently	0 (0)
Psychic stress due to pandemic	
None	223 (62.8)
Minor	101 (28.5)
Moderate	26 (7.3)
Major	5 (1.4)
Fear of negative effects on cancer therapy	
None	234 (65.9)
Minor	102 (28.7)
Moderate	16 (4.5)
Major	3 (0.8)

### Part C: mHealth App Use

About 1 out of 4 (n=83, 23%) participants expected no positive effect of digital health solutions on cancer care, whereas 1 out of 3 (n=114, 32%) expected minor effects. Only 19% (n=67) and 10% (n=34) of the participants anticipated moderate or major effects. Additionally, 16% (n=57) of the participants saw no need for digitalization in cancer care at all.

About 1 out of 4 participants (n=83, 23%) expected no positive effect of digital health solutions on cancer care, whereas 1 out of 3 (n=114, 32%) expected minor effects.

Only 10% (n=34) of participants were currently using health apps, with 8% (n=27) using only one health app and 2% (n=7) using more than one health app. Additionally, 6% (n=23) had formerly used a health app, whereas the majority of the participants (n=298, 84%) had never used a health app.

Out of the 57 (16%) app users, about half had been using lifestyle apps (eg, fitness trackers, nutrition, and exercise apps; n=28, 49%), and every fourth user had been using a service-oriented health app (eg, health insurance apps, apps for monitoring medication intake, scheduling doctor’s appointments, and symptom diaries, etc; n=14, 25%) or medical apps (eg, apps supporting diagnosis and therapy of diseases, such as blood sugar measurement, tinnitus treatment, and behavioral therapy; n=15, 26%).

About two-thirds of the participants expressed willingness to share their health data using a mHealth app. The most frequent possible recipients identified were researchers (n=135, 43%), therapists (n=134, 38%), and the participants themselves (n=112, 32%). Only a minority of participants indicated willingness to share their data for self-help purposes (n=58, 16%) or publicly (n=44, 12%).

Out of 355 participants, 151 (43%) indicated that they would never use a health app to record symptoms of their cancer. The responses varied among those willing to use such an app: 103 (29%) participants preferred to use it symptom-dependent rather than at a fixed frequency. Specifically, 38 (11%) participants would use it once, whereas 63 (18%) participants would use it frequently, with breakdowns as follows: every 3 months (n=15, 4%), monthly (n=15, 4%), weekly (n=15, 4%), multiple times weekly (n=8, 2%), daily (n=7, 2%), and multiple times daily (n=3, 1%).

Asked about their desired interaction within a cancer app, 145 out of 355 (41%) indicated that they would not use it. Among potential users, preferences for interaction varied: 158 (45%) participants expressed interest in interacting with physicians, whereas 98 (28%) participants wanted to interact with researchers. An even smaller proportion preferred interaction with nurses (n=41, 12%) or engaging in self-care activities (n=41, 12%). Only 15 (4%) participants stated they did not desire any interaction within the app.

In summary, a comprehensive summary of all results for Part C can be found in Table 3.

**Table 3.** Digital health attitudes and app usage patterns (see Table S1 in Multimedia Appendix 1 for the detailed overview of the questions).

Variable and category	Values, n (%)
Effect of digital health on cancer care	
No effect	83 (23.4)
Minor	114 (32.1)
Moderate	67 (18.9
Major	34 (9.6)
Not necessary	57 (16.1)
Health app usage	
Nonuser	298 (83.9)
Ex-user	23 (6.5)
Single-app user	27 (7.6)
Multiapp user	7 (2)
Type of health app used (n=57/355)	
No app	297 (83.9)
Lifestyle	28 (7.9)
Service	14 (4)
Medical	15 (4.2)
Willingness to share health data	
Public	44 (12.4)
Self-help groups	58 (16.3)
For personal use	112 (31.5)
Not at all	120 (33.8)
Therapists	134 (37.7)
Researchers	153 (43.1)
Intended frequency of app use (n=63/355)	
Multiple times daily	3 (0.8)
Daily	7 (2)
Multiple times weekly	8 (2.3)
3× monthly	15 (4.2)
Monthly	15 (4.2)
Weekly	15 (4.2
Once	38 (10.7)
Symptom-dependent	103 (29)
Never	151 (42.5)
Desired interaction within the cancer app	
Nobody	15 (4.2)
Self-care	41 (11.5)
Nurses	41 (11.5)
Researchers	98 (27.6)
No app use	145 (40.8)
Physicians	158 (44.5)

### Relationships Between Patient Characteristics and Study Outcomes

Furthermore, we explored how demographic variables correlated with survey responses. Depending on the measurement level of the variables, either Pearson (for continuous variables) or Spearman (for ordinal variables) correlations were applied, revealing several significant relationships: older age was negatively associated with app usage (*P*<.001, *r*=−0.20), psychological stress (*P*=.009, *r*=−0.14), and the expected effect of digital solutions on cancer care (*P*=.39, *r*=−0.11). In addition, we found the following:

- Male participants showed a negative correlation with fear of SARS-CoV-2 infection during (*P*=.03, *r*=−0.11) and after the pandemic (*P*=.03, *r*=−0.11).
- Higher education positively correlated with app usage (*P*<.001, *r*=0.17) and higher professional qualification with expected effect of digital solutions (*P*=.049, *r*=0.10). Moreover, a higher educational level was associated with more significant concern about pandemic-related impacts on personal cancer outcomes (*P*=.05, *r*=0.15).
- Perceived disruptions of cancer care due to the pandemic, fear of personal repercussions, and cancellation of appointments by the clinic (*P*<.001, *r*=0.39) or by the patient (*P*<.001, *r*=0.34) were all positively correlated with fear of SARS-CoV-2 infection and psychological stress (all *P*<.001, *r*=0.39; *P*<.001, *r*=0.59).
- The perceived impact of the pandemic on participants’ cancer care was positively correlated with app usage (*P*=.03, *r*=0.12).

To understand relationships between patient characteristics and study outcomes, we examined bivariate correlations between key variables. Table 4 presents all statistically significant correlations, organized to show how demographic factors (age, gender, education, and qualification), pandemic experiences (impact, fear, and cancellations), and mHealth attitudes (digital solutions, app usage, and frequency) relate to one another.

**Table 4.** Overview of all statistically significant Pearson and Spearman correlations^a^.

Variable 1 and variable 2		*P* value	*r*
Age			
	Gender	<.001	0.18^b^
	Education	<.001	−0.25^b^
	Employment	<.001	−0.64^c^
	Psychic stress	.009	−0.14^b^
	App usage	<.001	−0.20^b^
	Digital solution	.04	−0.11^b^
	Frequency	<.001	−0.24^b^
Gender			
	Fear before	.04	−0.11^b^
	Fear now	.04	−0.11^b^
Education			
	Qualification	<.001	0.33^d^
	Negative effects	.005	0.15^b^
	Digital solution	.07	0.10
	App usage	<.001	0.17^b^
	Frequency	<.001	0.24^b^
Qualification			
	Cancellation (clinic)	<.001	0.18
	Cancellation (patient)	.007	0.15
	Digital solution	.049	0.10
	App usage	.10	0.09
Impact			
	Fear before	<.001	0.39
	Fear now	<.001	0.31
	Cancellation (clinic)	<.001	0.43
	Cancellation (patient)	<.001	0.34
	Psychic stress	<.001	0.39
	Negative effects	<.001	0.45
	Digital solution	.10	0.09
	App usage	.03	0.12
Negative effects			
	Fear before	<.001	0.45
	Fear now	<.001	0.55
	Cancellation (clinic)	<.001	0.44
	Cancellation (patient)	<.001	0.35
	Psychic stress	<.001	0.59
	Digital solution	.03	0.11
	Frequency	.003	0.16

a Bivariate correlations were calculated between demographic characteristics, pandemic experiences, and mHealth attitudes. Depending on the measurement level of the variables, either Pearson (for continuous variables) or Spearman (for ordinal variables) correlations were used. Correlation coefficients (*r* or ρ) indicate the strength and direction of the relationship: weak correlations=0.10≤*r*≤0.29, moderate correlations=0.30≤*r*≤0.49, else strong correlation. Positive coefficients indicate that variables increase together, whereas negative coefficients indicate that one variable increases as the other decreases.

b Weak correlation.

c Strong correlation.

d Moderate correlation.

Note that it must be emphasized that correlations are not a robust measure of association and do not allow causal inference. Moreover, the analyses did not account for potential confounding variables.

## Discussion

### Overview of Findings

This study provides new insights into the experiences of patients with HNC in terms of diagnosis, treatment, and follow-up care, as well as their attitudes toward mHealth apps. Although most patients reported no major disruptions to their cancer treatment, a significant proportion (20%‐26%) experienced interruptions—particularly in follow-up appointments and treatment-related checkups. This subgroup highlights the importance of maintaining continuity of care for all patients, even during health care crises.

Correlation analyses revealed several notable patterns. Older participants were less likely to use digital health solutions, whereas higher levels of education were associated with greater acceptance of mHealth. In addition, patients who had experienced pandemic-related disruptions to care reported both increased anxiety and greater interest in digital alternatives. The observed correlations between pandemic-related anxiety, disruptions in care, and psychological distress (*r*=0.27‐0.49) suggest that these factors form an interconnected cluster of pandemic-related health care anxieties.

### Pandemic Impact on Cancer Care

The COVID-19 pandemic has posed unprecedented challenges to health care systems worldwide and necessitated rapid adjustments in cancer care [21]. At our institution, treatment pathways remained largely stable; however, 20%‐26% of patients reported interruptions in care, which is consistent with international observations of treatment delays, limited access to support services, and changed follow-up appointments [22,23]. Previous studies on head and neck oncology also document pandemic-related delays and increased psychological distress [23], while short-term survival outcomes appear to have remained largely unaffected [24]. These findings suggest that rapid institutional adjustments have effectively mitigated serious clinical consequences despite significant logistical and emotional challenges.

### Potential of Digital Health Solutions

Despite current low usage (10%), patients showed significant interest in mHealth solutions for symptom monitoring, communication with health care providers, and data sharing. Greater acceptance was associated with younger age, higher education level, and pandemic-related disruptions to care, consistent with general trends in telemedicine adoption during COVID-19 [25], although regulatory barriers remain in many countries [26].

mHealth offers opportunities for remote symptom monitoring, timely interventions, and personalized patient education. Feasibility in patients with HNC has been demonstrated in pilot studies [27] and randomized controlled trials [28]. However, large-scale implementation continues to be limited by early discontinuation, challenges with scalability, and limited integration into routine clinical workflows [29].

### Barriers to Adoption

The findings reveal several barriers to the adoption of mHealth that are consistent with previous research findings. Age, digital literacy, and perceived usefulness remain important determinants of acceptance [30]. Although most participants stated that they had minimal concerns about data security within the university, previous studies show that data protection outside clinical research environments remains a major barrier [31,32]. This is particularly relevant in Germany, where data protection concerns are often cited as a significant barrier to the adoption of digital health services [32].

### Implications for Practice

Future strategies to enhance mHealth adoption in HNC care should include the following:

1. Improving digital literacy—targeted training programs for older adults and individuals with lower educational levels [33].
2. Addressing privacy concerns—transparent communication of robust data security measures [31].
3. Demonstrating utility—evidence-based, user-friendly, and personalized app functionalities to enhance trust and sustained use [34].

Previous studies suggest that despite ongoing concerns about implementation, oncologists are generally open to integrating mHealth technologies into patient care [35]. By removing barriers on both the patient and provider sides, mHealth could make the transition from a complementary tool to an integral part of multidisciplinary cancer treatment.

Although the COVID-19 pandemic was unprecedented, the challenges it revealed—such as scheduling constraints, communication gaps between visits, the need for remote monitoring, and increased patient anxiety about accessing health care—are not unique to pandemics. Similar disruptions also occur during natural disasters, staff shortages, transportation barriers, and routine capacity constraints. Our findings that 57% (n=202) of patients were willing to share health data and 45% (n=160) preferred to communicate with their doctors using an app provide actionable insights for strengthening continuity of care under various disruptive conditions.

The rapid digital transformation triggered by COVID-19 has permanently changed the expectations of patients and providers. The pandemic has accelerated a decade of digital health development in a matter of months, revealing both barriers and enablers that will continue to be relevant as health systems drive this transformation forward. The demographic predictors identified in our study—age, education, and prior experience with health care—represent enduring factors that are likely to influence mHealth adoption regardless of future circumstances.

### Strengths and Limitations

This study focused on a high-need population—patients with HNC—who often face complex treatments and intensive follow-up care requirements. By examining both pandemic-related disruptions to care and the introduction of digital health solutions within the same cohort, it offers a holistic perspective on health care resilience.

However, there are some limitations to consider. The study design, conducted at a single center in Würzburg, limits generalizability, and the relatively small, specialized cohort may not reflect the broader cancer population. Nonresponse bias cannot be ruled out, as the participation of nonrespondents was not tracked. The use of self-reported data may have led to bias due to recall or social desirability, although the survey design aimed to minimize these effects. The low prevalence of mHealth users (34/355, 10%) limited subgroup analyses and restricts the generalization of user behavior. Finally, correlation analyses do not allow conclusions about causality and did not control for potential confounding factors. Future multicenter studies with larger, more diverse samples and causal modeling approaches are needed to validate and extend these findings.

### Conclusion

This study highlights both the challenges and opportunities faced by patients with HNC during and after the COVID-19 pandemic. While most patients continued to have access to essential care, a subset experienced significant disruptions, underscoring the need for resilient care pathways. The pandemic has also sparked interest in mHealth apps; however, barriers remain in terms of age, digital literacy, and perceived usefulness. Targeted education, robust data protection, and clear evidence of clinical benefit will be critical to integrating mHealth into cancer care and improving outcomes and quality of life for patients with HNC.

## Supplementary material

10.2196/65192 Multimedia Appendix 1 Table S1. Complete cancer and COVID-19 survey within the Corona Health app.

10.2196/65192 Checklist 1 STROBE checklist.

## Abbreviations

HNC: head and neck cancer
mHealth: mobile health
STROBE: Strengthening the Reporting of Observational Studies in Epidemiology
