# Supplementary material for: Exploring the Adoption of Mobile Health Apps Among Patients with Head and Neck Cancer After the COVID-19 Pandemic: Secondary Analysis of Cross-Sectional Survey
Source: JMIR Cancer. 2026 Apr 15;12:e65192. doi: 10.2196/65192 (PMC13082343; doi:10.2196/65192)
Supplement: Multimedia Appendix 1 [file cancer-v12-e65192-s001.docx]

| **Cancer and Covid-19 survey** | | |
| --- | --- | --- |
| Q# | **Variable name** | **Question** |
| **Part A (sociodemographic information)** | | |
| 1 | age | How old are you (in years)? |
| 2 | age diagnosis | How old were you (in years) when you were diagnosed with cancer? |
| 3 | gender | Which gender do you classify yourself as? |
| 4 | entity | What is the origin of your cancer? |
| 5 | country | In which country was or is your cancer being treated? |
| 6 | living situation | Do you live together with your family and do they support you in coping with your cancer? |
| 7 | education | How many years of education do you have (not including vocational training/university studies)? |
| 8 | qualification | How many years of professional training and/or university studies do you have (not including time spent in school)? |
| 9 | employment | What is your current professional situation? |
| **Part B (impact of the pandemic on cancer)** | | |
| 10 | disease status | What was your situation during the first years of the pandemic in 2020 and 2021 regarding your cancer (multiple answers possible)? |
| 11 | therapy | How was or is your cancer treated (multiple answers possible)? |
| 12 | impact | Has your cancer treatment or follow-up been affected by the COVID 19 pandemic? |
| 13 | impact therapy | If treatment was affected, what type of treatment for your cancer was affected by the COVID 19 pandemic (multiple answers possible)? |
| 14 | fear before | Were you afraid of contracting the coronavirus in the past when attending appointments for treatment or follow-up care for your cancer? |
| 15 | fear now | Are you currently afraid of contracting the coronavirus when you attend appointments for treatment or follow-up care for your cancer? |
| 16 | cancellation clinic | How often were appointments for treatment or follow-up care of your cancer canceled or postponed by the clinic or doctor's office? |
| 17 | cancellation patient | How often have you yourself canceled appointments for treatment or follow-up care for your cancer? |
| 18 | psychic stress | Have you noticed any psychological effects on yourself as a result of the COVID 19 pandemic (e.g., sleep disorders, anxiety, depression)? |
| 19 | negative effects | Are you concerned that the COVID 19 pandemic could have a negative impact on the success of your cancer treatment or follow-up (e.g., later detection of cancer recurrence due to postponed examinations)? |
| **Part C (mHealth application use)** | | |
| 20 | digital solution | Do you think that any negative impact that the COVID 19 pandemic has on the care of cancer patients could be avoided or reduced by digitising the healthcare system? |
| 21 | app usage | Have you already used and/or are you currently using one or more health apps? |
| 22 | app type | What type(s) of health apps have you used or do you currently use (multiple answers possible)? |
| 23 | health data | For what purpose would you be prepared to make health data (e.g., about your cancer) available via an app (multiple answers possible)? |
| 24 | frequency | How often would you be willing to use a health app (e.g., to record symptoms of your cancer)? |
| 25 | interaction | Who would you contact via the app if, for example, complaints requiring clarification were entered (multiple answers possible)? |

Table 1: Complete Cancer and Covid-19 survey within the Corona Health App. The survey includes sociodemographic information of the participants (part A), the impact of the pandemic on their diagnosis, treatment, and aftercare (part B), as well as their interest in and willingness to use mobile Health (mHealth) applications (part C).
